# Association of perceived work pace and physical work demands with occupational accidents: a cross-sectional study of ageing male construction workers in Denmark

**DOI:** 10.1186/s12889-021-12461-6

**Published:** 2022-01-06

**Authors:** Pernille Weber Hansen, Vivi Schlünssen, Kirsten Fonager, Jakob Hjort Bønløkke, Claus D. Hansen, Henrik Bøggild

**Affiliations:** 1 Department of Public Health, Environment, Occupation and Health, Danish Ramazzini Centre, Aarhus University, Bartholins Allé 2, 8000 Aarhus C, Denmark; 2 Department of Clinical Medicine, Aalborg University, Sdr. Skovvej 15, 9000 Aalborg, Denmark; 3 National Research Centre for the Working Environment, Lersø Parkallé 105, 2100 Copenhagen Ø, Denmark; 4 Department of Social Medicine, Aalborg University Hospital, Havrevangen 1, 9000 Aalborg, Denmark; 5 Department of Occupational and Environmental Medicine, Danish Ramazzini Centre, Aalborg University Hospital, Havrevangen 1, 9000 Aalborg, Denmark; 6 Department of Sociology and Social Work, Aalborg University, Fibigerstræde 13, 9220 Aalborg Ø, Denmark; 7 Department of Health Science and Technology, Public Health and Epidemiology Group, Aalborg University, Niels Jernes Vej 14, 9220 Aalborg, Denmark

**Keywords:** Blue-collar worker, Job demand, Manual worker, Work accident, Work characteristic

## Abstract

**Background:**

Occupational accidents continue to be a significant public health challenge worldwide. Construction workers in particular are at high risk of occupational accidents, and thus it is of major importance to identify possible predictors of occupational accidents among construction workers. We aimed to investigate the association between self-reported work pace and physical work demands and occupational accidents among ageing male construction workers in Denmark.

**Methods:**

Data on perceived work pace, physical work demands, and occupational accidents was acquired from questionnaires sent to ageing construction workers in Denmark in 2016 as part of the ALFA project (ALdring og Fysisk Arbejde; Ageing and Physical Work). A sample of 1270 Danish male construction workers above 50 years of age was included in the present study. Multiple logistic regression models were applied, with adjustments for age, smoking, body mass index, musculoskeletal disorders, occupation, work experience, and support at work.

**Results:**

Of 1270 construction workers, 166 (13.1%) reported an occupational accident within the last 12 months. There was no significant association between perceived work pace and occupational accidents, but physical work demands were associated with higher odds for occupational accidents, with an odds ratio of 2.27 (95% confidence interval 1.26–4.10) for medium physical work demands and 2.62 (95% confidence interval 1.50–4.57) for high physical work demands.

**Conclusions:**

Ageing male construction workers with high physical work demands had statistically significant higher odds of having an occupational accident. By contrast, perceived work pace was not associated with occupational accidents in this large cross-sectional study.

**Supplementary Information:**

The online version contains supplementary material available at 10.1186/s12889-021-12461-6.

## Background

Occupational accidents continue to be a significant public health challenge worldwide. In 2014, approximately 374 million workers had an occupational accident requiring at least 4 days of absence, while 380,500 workers died due to an accident at work [1]. Despite efforts to reduce occupational accidents through preventive measures over the past decades, the prevalence of occupational accidents in Denmark is still high, with more than 42,000 non-fatal and fatal occupational accidents each year [2, 3]. This results in a great economic burden on society from additional health care expenses and premature retirements from the workforce [4, 5], in addition to severe health, social, and economic consequences for the affected workers, their families, and workplaces [6].

In particular, construction workers have a higher prevalence and risk of occupational accidents than other occupations [3, 7–9] probably due to their differential exposure to hazardous environments, equipment, and tasks in daily work [8, 9]. The need to prevent occupational accidents among construction workers is thus evident.

The construction industry is characterised by high work pace and physically demanding work [10]. Several studies have demonstrated that high physical and psychological job demands are associated with a higher risk of occupational accidents [7, 8, 11–15]. An explanation may be that physical work demands can generate fatigue [16, 17] and decrease the ability to process information and adequately react to a dangerous situation [18]. Furthermore, some physically demanding tasks among construction workers may themselves be riskier than less demanding tasks.

In addition, studies have found that job stress is related to higher odds of occupational accidents among building construction workers and coal miners [19, 20].

However, a limitation of these studies is that they have combined work pace and physical work demands in one exposure measure, making it difficult to identify the specific factors associated with occupational accidents, which is critical for evidence-based prevention of occupational accidents. Only one of these studies has assessed the specific relationship between work pace, physical work demands, and occupational accidents, indicating work pace and physical work demands to be associated with a higher risk of an accident [8]. Other factors at work, e.g. noise, shift work, and lack of access to assistive devices, as well as individual factors such as sleep disturbances or alcohol use may also contribute to the risk of occupational accidents [21, 22]. Physical work ability required in building and construction work, e.g. strength and speed, decreases with age [23, 24]. Accordingly, younger workers may be less affected by work pace and physical work demands than older workers, who may be less able to withstand the demands. Experience may, on the other hand, protect older workers from the risks of accidents with a high work pace and physical work demands.

Although a study has examined the relationship between work-related factors and occupational accidents among ageing workers [25], knowledge about how work factors are related to occupational accidents, specifically among ageing construction workers, remains limited.

We investigated the hypothesis that work pace and physical work demands were associated with higher odds of an occupational accident among ageing construction workers.

## Methods

### Population

**Fig. 1:**
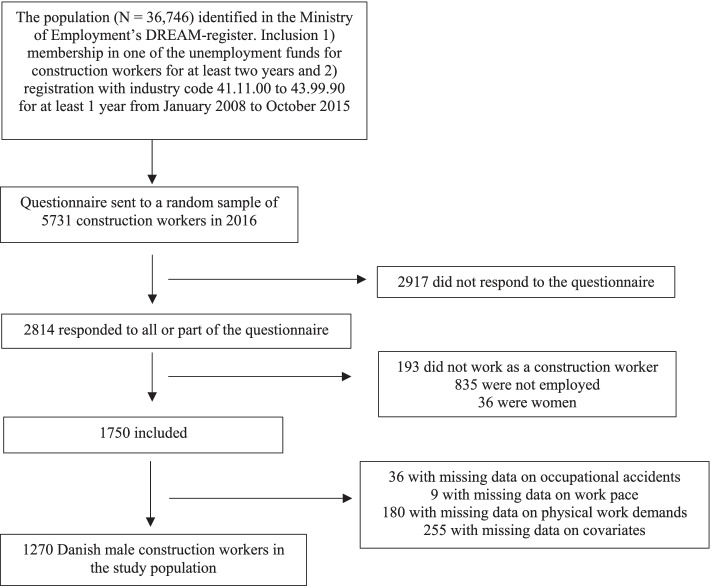
Flowchart of the study population

Data for this cross-sectional study were acquired from questionnaires sent to ageing Danish construction workers as part of the ALFA project (ALdring og Fysisk Arbejde; Ageing and Physical Work) [26]. In 2016, a random sample of 5731 Danish construction workers born before 1967 received a questionnaire regarding health, work environment, and attitudes toward retirement. Of them, 2814 responded to all or part of the questionnaire (response rate, 49%). Respondents who answered that they did not work as a construction worker or were not employed were excluded from the analyses. Female respondents were also excluded due to a small proportion of women. In addition, respondents who had not answered all questions related to outcome, exposures, and covariates were excluded from the analyses, resulting in a sample of 1270 Danish male construction workers (Fig. 1).

### Occupational accidents – outcome

An occupational accident was defined as ‘a discreet, sudden and unexpected incident during work leading to physical or mental injury’ [27]. We used a question originally from The Work Environment and Health study [28]: ‘Have you been involved in one or more occupational accidents within the last 12 months?’ and dichotomised the answer into ‘No’, including those who answered ‘No accidents’, and ‘Yes’, including those who answered ‘One accident’, ‘Two accidents’, ‘Three accidents’, or ‘Four or more accidents’.

### Perceived work pace – exposure

Perceived work pace was measured using two questions retrieved from Copenhagen Psychosocial Questionnaire (COPSOQ-II) [29] related to the respondents’ work in general: ‘Is it necessary to work very fast?’ and ‘Is the work pace high throughout the whole workday?’ They were combined into a scale. This was considered acceptable, with a Cronbach’s alpha of 0.77 [30]. Three groups were created based on the respondents’ answers to the two questions. Respondents who answered ‘Always’ or ‘Often’ were categorised as having high work pace; ‘Sometimes’ were categorised as having medium work pace; and ‘Rarely’ or ‘Never/hardly never’ had low work pace.

### Physical work demands – exposure

Physical work demands were measured using a question acquired from The Work Environment and Health study [28]: ‘How physically demanding do you usually perceive your current work?’ with a numeric 11-point response scale ranging from 0 (‘not hard’) to 10 (‘maximally hard’). Three exposure groups were created, and the cutoff points were chosen based on the definitions of the scale and to have a reasonable distribution of respondents. Respondents who rated <=5 on the scale were categorised as low physical work demands; 6 or 7, medium physical work demands; and > =8, high physical work demands.

### Covariates

Putative confounders were identified a priori by reviewing the literature and using Directed Acyclic Graphs [31]. Age, body mass index, smoking, and musculoskeletal disorders were selected for adjustment due to their association with occupational accidents [7, 8, 13, 32–34] and association with work ability as well as health status [26, 35–38]. Occupation, work experience, and support at work were included as work-related covariates as they have been identified as relevant risk factors for occupational accidents in other studies [8, 12, 34, 39, 40] and are assumed to be associated with the exposure of interest. *Age* was categorised into four age groups: 50–54 years, 55–59 years, 60–64 years, and 65+ years. *Body Mass Index* (BMI) was calculated as weight (kg)/height(m)^2^ based on self-reported weight and height, and categorised into BMI ≤ 24.9, 25–29.9, and ≥ 30. *Smoking* was measured using the question ‘Do you smoke?’ The respondents who smoked daily or sometimes were categorised as current smokers, those who used to smoke but not anymore were categorised as former smokers, and those who had never smoked were categorised as never smokers. *Musculoskeletal disorder* was measured using the question ‘Do you have any disorders in the following parts of the body (Yes/No) – neck/shoulders, back, upper extremities, and lower extremities?’ *Musculoskeletal disorder* was assessed for all four body regions separately. *Occupation* was identified by asking the respondents to state their job title, which was subsequently coded according to the Danish version of the International Standard Classification of Occupations (DISCO-08). The four most frequent occupations were categorised according to their respective titles Carpenters and Joiners, Building and Related Electricians, Plumbers and Pipe Fitters, and Painters and Related Workers, whereas the remaining occupations were aggregated and categorised as Others. *Work experience* was measured using the question ‘How many years have you worked in your current field?’ and categorised into four groups based on the quartiles: 0–22 years, 23–34 years, 35–39 years, and 40–64 years. *Support at work* was measured using the question ‘How often do you get help and support from your colleagues?’ and categorised into high, medium, and low. Respondents who answered ‘Always’ and ‘Often’ were categorised as having high support at work; ‘Sometimes’, medium support at work; and ‘Rarely’ and ‘Never/hardly never’, low support at work.

### Statistical analyses

Simple descriptive statistics were used to describe the characteristics of the study population in frequencies and percentages.

Multiple logistic regression models were used to estimate odds ratios (OR) and 95% confidence intervals (CI). All analyses were conducted with *occupational accidents* as the dependent variable and *work pace* or *physical work demands* as the independent variable. Both independent variables had the category ‘Low’ as the reference. In addition to crude logistic regression models, adjusting for each of the covariates were conducted, and as only modest changes were seen after adjustment, two models were estimated. Model 1 was adjusted for age, BMI, smoking, and musculoskeletal disorders (demographic, lifestyle, and health characteristics); Model 2 was further adjusted for occupation, work experience, and support at work (work characteristics). Additionally, a mutual-adjusted logistic regression was conducted to assess the concurrent effect of both exposures.

Pearson chi-square tests were performed to compare respondents with non-respondents and compare those included in the final study population with those excluded due to incomplete data (*non-response analyses*). The comparison between the groups was made using register data. The groups were compared in terms of age, residence region, family income, and the number of children.

A *p*-value of < 0.05 was considered statistically significant. All analyses were conducted using Stata 15.1 (StataCorp LLC, College Station, TX, USA).

## Results

Of the respondents, 166 construction workers (13.1%) had an occupational accident within the last 12 months. Most construction workers were aged 50–54 years (35.7%) or 55–59 years (35.3%) (Table 1). 23% were current smokers, and 18.3% had a BMI of 30.0 or above. Most construction workers were carpenters and joiners (23.1%) and had 40–64 years of work experience in their current occupation (28.7%). 31.4% had musculoskeletal disorders in their lower extremities.

**Table 1:** Characteristics of the study population (*N* = 1270), divided by exposure. Number (percentage)

		Work pace			Physically demanding work		
	All	Low	Medium	High	Low	Medium	High
Age							
50–54 years	453 (35.7)	35 (28.9)	196 (38.1)	222 (35.0)	96 (35.0)	140 (36.5)	217 (35.5)
55–59 years	449 (35.3)	33 (27.3)	168 (32.7)	248 (39.1)	78 (28.5)	124 (32.3)	247 (40.4)
60–64 years	279 (22.0)	36 (29.8)	116 (22.6)	127 (20.0)	70 (25.6)	97 (25.3)	112 (18.3)
65+ years	89 (7.0)	17 (14.1)	34 (6.6)	38 (6.0)	30 (11.0)	23 (6.0)	36 (5.9)
BMI							
≤ 24.9	394 (31.0)	35 (29.0)	170 (33.1)	189 (29.8)	77 (28.1)	118 (30.7)	199 (32.5)
25.0–29.9	644 (50.7)	59 (48.8)	248 (48.3)	337 (53.1)	139 (50.7)	188 (49.0)	317 (51.8)
≥ 30.0	232 (18.3)	27 (22.3)	96 (18.7)	109 (17.2)	58 (21.2)	78 (20.3)	96 (15.7)
Smoking							
Never	519 (40.9)	47 (38.8)	225 (43.8)	247 (38.9)	125 (45.6)	151 (39.3)	243 (39.7)
Former	459 (36.1)	49 (40.5)	178 (34.6)	232 (36.5)	100 (36.5)	142 (37.0)	217 (35.5)
Current	292 (23.0)	25 (20.7)	111 (21.6)	156 (24.6)	49 (17.9)	91 (23.7)	152 (24.8)
Musculoskeletal disorders							
Neck/shoulders	348 (27.4)	30 (24.8)	132 (25.7)	186 (29.3)	66 (24.1)	94 (24.5)	188 (30.7)
Back	358 (28.2)	39 (32.2)	126 (24.5)	193 (30.4)	70 (25.6)	105 (27.3)	183 (29.9)
Upper extremities	239 (18.8)	15 (12.4)	83 (16.2)	141 (22.2)	37 (13.5)	63 (16.4)	139 (22.7)
Lower extremities	399 (31.4)	37 (30.6)	155 (30.2)	207 (32.6)	77 (28.1)	128 (33.3)	194 (31.7)
Occupation							
Carpenters and Joiners	293 (23.1)	24 (19.8)	117 (22.8)	152 (23.9)	53 (19.3)	95 (24.7)	145 (23.7)
Building and Related Electricians	169 (13.3)	17 (14.1)	89 (17.3)	63 (9.9)	51 (18.6)	74 (19.3)	44 (7.2)
Plumbers and Pipe Fitters	96 (7.5)	5 (4.1)	37 (7.2)	54 (8.5)	6 (2.2)	33 (8.6)	57 (9.3)
Painters and Related Workers	58 (4.6)	2 (1.7)	19 (3.7)	37 (5.8)	9 (3.3)	20 (5.2)	29 (4.7)
Others	654 (51.5)	73 (60.3)	252 (49.0)	329 (51.8)	155 (56.6)	162 (42.2)	337 (55.1)
Work experience							
0–22 years	308 (24.3)	47 (38.8)	125 (24.3)	136 (21.4)	92 (33.6)	79 (20.6)	137 (22.4)
23–34 years	324 (25.5)	32 (26.5)	131 (25.5)	161 (25.4)	58 (21.2)	103 (26.8)	163 (26.6)
35–39 years	273 (21.5)	13 (10.7)	101 (19.7)	159 (25.0)	40 (14.6)	82 (21.4)	151 (24.7)
40–64 years	365 (28.7)	29 (24.0)	157 (30.5)	179 (28.2)	84 (30.7)	120 (31.3)	161 (26.3)
Support at work							
Low	223 (17.6)	25 (20.7)	77 (15.0)	121 (19.1)	44 (16.1)	63 (16.4)	116 (19.0)
Medium	494 (38.9)	34 (28.1)	209 (40.7)	251 (39.5)	116 (42.3)	145 (37.8)	233 (38.1)
High	553 (43.5)	62 (51.2)	228 (44.4)	263 (41.4)	114 (41.6)	176 (45.8)	263 (43.0)
Work pace							
Low	121 (9.5)	–	–	–	60 (21.9)	35 (9.11)	26 (4.3)
Medium	514 (40.5)	–	–	–	138 (50.4)	200 (52.1)	176 (28.8)
High	635 (50.0)	–	–	–	76 (27.7)	149 (38.8)	410 (67.0)
Physical work demands							
Low	274 (21.6)	60 (49.6)	138 (26.9)	76 (12.0)	–	–	–
Medium	384 (30.2)	35 (28.9)	200 (38.9)	149 (23.5)	–	–	–
High	612 (48.2)	26 (21.5)	176 (34.2)	410 (64.6)	–	–	–
Occupational accident							
No	1104 (86.9)	108 (89.3)	446 (86.8)	550 (86.6)	257 (93.8)	333 (86.7)	514 (84.0)
Yes	166 (13.1)	13 (10.7)	68 (13.2)	85 (13.4)	17 (6.2)	51 (13.3)	98 (16.0)

*BMI*: Body Mass Index (kg/m^2^)

In the crude and adjusted analysis, there was no statistically significant association between perceived work pace and occupational accidents in any of the models (Table 2). In contrast to perceived work pace, there was a statistically significant association between physical work demands and occupational accidents in all models (Table 3). In the fully adjusted Model 2, odds of an occupational accident were 2.27 (95%CI 1.26–4.10) for construction workers with medium physical work demands and 2.62 (95%CI 1.50–4.57) times higher for construction workers with high physical work demands compared with construction workers with low physical work demands.

**Table 2:** Association between work pace and occupational accidents, unadjusted and adjusted, odds ratios (OR) and 95% confidence intervals (CI)

	N	Crude	Model 1^a^	Model 2^b^
	Case/Exposed	OR (95% CI)	OR (95% CI)	OR 95% CI
Work pace				
Low	13/121	1.00 (ref)	1.00 (ref)	1.00 (ref)
Medium	68/514	1.27 (0.67–2.38)	1.24 (0.65–2.37)	1.35 (0.70–2.60)
High	85/635	1.28 (0.69–2.38)	1.19 (0.63–2.27)	1.29 (0.67–2.48)

^a^ Model 1 adjusts for age, body mass index, smoking, and musculoskeletal disorders

^b^ Model 2 adjusts for age, body mass index, smoking, musculoskeletal disorders, occupation, work experience and support at work Ref: Reference group

**Table 3:** Association between physical work demands and occupational accidents, unadjusted and adjusted, odds ratios (OR) and 95% confidence intervals (CI)

	N	Crude	Model 1^a^	Model 2^b^
	Case/Exposed	OR (95% CI)	OR (95% CI)	OR 95% CI
Physical work demands				
Low	17/274	1.00 (ref)^*^	1.00 (ref)	1.00 (ref)
Medium	51/384	**2.32 (1.31–4.10)**	**2.28 (1.27–4.07)**	**2.27 (1.26–4.10)**
High	98/612	**2.88 (1.69–4.93)**	**2.70 (1.56–4.67)**	**2.62 (1.50–4.57)**

^a^ Model 1 adjusts for age, body mass index, smoking, and musculoskeletal disorders

^b^ Model 2 adjusts for age, body mass index, smoking, musculoskeletal disorders, occupation, work experience and support at work. Statistically significant results (*p* < 0.05) are marked in bold. Ref: Reference group

Further, the mutually adjusted analysis, Table 4, indicated that adjusting for perceived work pace did not change the association between physical work demands and occupational accidents (compared with the estimates from Table 3, crude and Model 2), while the small effect of perceived work pace on accidents (Table 2, crude and Model 2) disappeared, suggesting that the physical work demands were responsible for the associations.

**Table 4:** Association between both work pace and physical work demands, results from the mutually adjusted logistic regression analysis, odds ratios (OR) and 95% confidence intervals (CI)

	Crude^a^	Model 1^b^
	OR 95% CI	OR 95% CI
Work pace		
Low	1.00 (ref)	1.00 (ref)
Medium	1.03 (0.54–1.97)	1.12 (0.58–2.19)
High	0.88 (0.46–1.67)	0.94 (0.48–1.85)
Physical work demands		
Low	1.00 (ref)	1.00 (ref)
Medium	**2.35 (1.32–4.19)**	**2.28 (1.26–4.13)**
High	**3.06 (1.75–5.35)**	**2.73 (1.53–4.88)**

^a^ the total impact of work pace and physical work demands on occupational accidents adjusted for each other

^b^ the total impact of work pace and physical work demands on occupational accidents adjusted for each other as well as adjusted for age, body mass index, smoking, musculoskeletal disorders, occupation, work experience, and support at work

Statistically significant results (*p* < 0.05) are marked in bold. Ref: Reference group

Younger construction workers, construction workers living in the Capital Region of Denmark or Region Zealand, and construction workers with a lower family income were more likely to be non-respondents (Supplementary Table S1). Older construction workers were more inclined to have incomplete data.

## Discussion

In this study, perceived work pace was not significantly associated with occupational accidents. However, there was an association between physical work demands and occupational accidents among ageing male construction workers, even after adjustment for relevant covariates.

No earlier studies have investigated the specific association between work pace and occupational accidents. However, some studies have examined the closely related exposure of time pressure. In line with our study, Rasmussen et al. [41] found no association between time pressure and occupational accidents among Danish adolescents. By contrast, Van der Klauw et al. [42] showed that in the construction industry in the Netherlands, high time pressure was significantly associated with occupational accidents.

Studies have investigated work pace in combination with other work environment factors, showing job stress as well as high psychological job demands, including high work pace, to be associated with higher odds of an occupational accident [7, 8, 11–14, 19, 20, 25]. Chau et al. found that workers exposed to high psychological demands, including high work pace and mental load, had 1.35 (95%CI 1.02–1.78) times higher odds of an occupational accident compared with unexposed workers in a broad group of workers [7]. A crude odds ratio indicated that work pace was a significant risk factor for occupational accidents (OR 1.81 95%CI 1.35–2.41) in a broad group of workers [8]. Kiconco et al. similarly reported that a measure of job stress based on 14 items including work pace, was significantly associated with occupational accidents specifically among building construction workers (aPR 1.72 95%CI 1.22–2.41) [19]. Our finding supports that of Juliá et al., who combined work pace with five other work factors and found no association between this measure and occupational accidents among common male workers in Spain [40].

The inconsistent findings might be explained by the different exposure variables, as work pace in the present study is examined independently and not in combination with other work factors. Another explanation could be the categorisations of work pace as well as different study populations.

The finding that physical work demands were associated with occupational accidents is consistent with earlier studies exploring physical work demands in combination with other work factors [8, 11–15, 19, 20, 25]. For example, a cross-sectional study by Sakurai et al. showed that high job demands, measured from seven work factors including physical work demands, was significantly associated with occupational accidents among common workers in Japan (OR 1.44, 95%CI 1.28–1.63) [15]. Another large cross-sectional study among the general working population in France found that male workers exposed to high psychological demands had a 1.38 (95%CI 1.16–1.64) times higher odds of an occupational accident [11]. Moreover, Chau et al. estimated a crude OR of 3.37 (95%CI 2.47–4.61), suggesting that physical work demands were a strong risk factor for occupational accidents [8]. Finally, an earlier study among Danish adolescents found that high physical work demands were related to a 2.3-fold higher odds of an occupational accident [41] and Baidwan et al. found that ageing workers who perceived their workplaces to have high work demands had a risk nearly two times greater for occupational accidents [25]. We categorized physical work demands into three categories. Only one previous cohort study has applied the same categorisation, but did not identify the same associations, as only high and not medium psychologic demands were associated with significantly higher odds of an accident [14]. This contradictory finding may be explained by differences in the exposure variables.

### Strengths and limitations

A strength of the present study is that it investigates how specific work factors are associated with occupational accidents. Another strength is the thorough adjustment for putative confounding variables and that the results are consistent across different models. Also, the ALFA project was not designed to examine the associations between work pace, physical work demands, and occupational accidents, reducing the risk of selection and information bias due to knowledge among the respondents to the purpose of the study. There are also some limitations. First, the cross-sectional design precluded the determination of causal relationships. Hence, reverse causality cannot be ruled out, and being subject to an occupational accident may increase the likelihood of experiencing high physical work demands. In addition, we can also not rule out the possibility that people due to an accident (within the last 12 months) have got another job and other job demands. The questionnaire was concerned about accidents but did not distinguish whether the respondent had an injury or was unaffected by the accident. It would also have been relevant to know the specific type of accident and the seriousness, as minor injuries and more serious ones may be remembered differently when asked to remember a year back. It would, however, probably not be related to work pace or physical work demands and by that would underestimate the associations. Second, the study is based solely on self-reported data, which is vulnerable to reporting bias due to respondents’ selective reporting by outcome status or emotions [31]. For instance, it is not inconceivable that construction workers who have had an occupational accident are searching for a cause and therefore may report a higher work pace and physical work demands than construction workers without an accident at work, even though they have objectively had an equally high work pace and physical work demands. This would lead to differential misclassification and bias away from null. It is also possible that some of the workers have non-manual functions (foremen, office) and thus lower prevalence of physical work demands, but this should not raise estimates as it would probably be non-differential misclassification, as the registration of accidents should not be affected by having non-manual functions. The study included a heterogeneous group of occupations labelled as “others”. Their work environment may differ and the adjustment could leave residual confounding. Third, recall bias may have occurred as the respondents were asked to recall occupational accidents within the last 12 months, which may have led to an underestimation of occupational accidents, but probably not related to the work environment and thus without biasing the estimates. The timing of the exposures was not defined directly but related to the current work situation. Fourth, while the questions about work pace originate from a reliable and validated questionnaire (COPSOQ-II) [29], the questions concerning physical work demands and occupational accidents come from a large national questionnaire that is not validated (e.g. against registers or other questionnaires) [28]. However, it is still reasonable to assume that the respondents provided correct and accurate information as the questionnaire was developed based on research-based questions and methods as well as frequently used to identify health status and evaluate work environments in Denmark [28]. Although, minor accidents may have been underreported, as the question about occupational accidents is relatively unspecific in relation to severity. In addition, measuring solely *perceived* work pace may not grasp the actual objective pace of the work because experienced workers such as those participating in this study will have naturalised the pace with which they work. For that reason, the contrast in the variable will be low (which could be seen in Table 1 as a tendency to a ceiling effect) and it might not reflect differences in actual work pace but rather the perception of pace, which may be affected by many other aspects than the actual work pace.

Thus, more objective measures would have strengthened this study. Fifth, there was a low participation rate (49.1%) in the present study compared with other cross-sectional studies in the field [11, 12, 15]. A non-response analysis revealed some differences between respondents and non-respondents in terms of age, family income, and residence region. However, this is not expected to have led to selection bias and thereby influenced the associations in the study, as it seems unlikely that the respondents’ decision not to participate was dependent on both the exposure and outcome. Sixth, despite the adjustments made in the statistical analyses, residual confounding from other contributing factors that we did not have data about, cannot be excluded. Examples could be workplace noise, sleep disturbances, and alcohol use. For other work-related factors like psychological demands and social support from leaders, they could also be related to work accidents, but adjusting for these factors did not change estimates much (data not shown) and might lead to over adjustment of the results. The regression models were adjusting for up to seven potential confounders and with 166 outcomes, of which only 17 in the reference group for work demands, this could in theory lead to unstable models. We first examined the changes for each confounder individually in order to evaluate that adjustment did not dramatically change estimates compared to the unadjusted models, and the changes in estimates and confidence intervals were all less than 10%, and although cells with few accidents were present in the regression models, we found that the models remained stable. Finally, the differences between respondents and non-respondents and the possible bias may weaken the external validity of the study, as the respondents cannot be considered fully representative of all male ageing construction workers in Denmark.

## Conclusions

This study found that ageing construction workers having medium or high physical work demands had statistically significant higher odds of having had an occupational accident within the last 12 months compared with those reporting low physical work demands. By contrast, work pace was not related to occupational accidents. This indicates that physical work demands are a significant risk factor for occupational accidents, whereas work pace does not appear to be so, at least in this specific group of workers. However, due to methodological weaknesses related to the cross-sectional design, the small number of accidents and the self-reporting of both working environment and accidents, results should be interpreted with caution. Prospective studies using objective measures on work pace and demands as well as objective registrations of accidents are needed to guide future prevention strategies for work accidents.

## Supplementary Information

**Additional file 1: Table S1.** Results of the non-response analysis between respondents and non-respondents presented as frequencies and percentages N (%).

## Data Availability

As the study include sensitive information, restrictions apply to the availability of data that is not publicly available. The datasets used to analyse in the current study are available at the corresponding author on reasonable request.

## References

[1] Hämäläinen P, Takala J, Kiat TB (2017). Global estimates of occupational injuries and work-related illnesses 2017.

[2] Nielsen KJ, Carstensen O, Working Environment Council (2016). Videngrundlaget vedrørende omfang og karakter af arbejdsulykker [Knowledge about the extent and character of work accidents]. Rapport om arbejdsulykker og forebyggelse [Report on work accidents and prevention].

[3] Danish Working Environment Authority (2019). Arbejdstilsynets årsopgørelse 2018: Anmeldte arbejdsulykker 2013–2018 [Danish Working Environment Authority annual report 2018: reported work accidents 2013–2018].

[4] International Labour Office (2014). Safety and health at work: a vision for sustainable prevention: XX world congress on safety and health at work 2014: global forum for prevention 24–27 august 2014 Frankfurt, Germany/international labour office.

[5] Poulsen OM, Fridriksson JF, Tómasson K, Midtsundstad T, Mehlum IS, Hilsen AI (2017). Working environment and work retention.

[6] Dembe AE (2001). The social consequences of occupational injuries and illnesses. Am J Ind Med.

[7] Chau N, Lemogne C, Legleye S, Choquet M, Falissard B, Fossati P (2011). Are occupational factors and mental difficulty associated with occupational injury?. J Occup Environ Med.

[8] Chau N, Bourgkard E, Bhattacherjee A, Ravaud JF, Choquet M, Mur JM (2008). Associations of job, living conditions and lifestyle with occupational injury in working population: a population-based study. Int Arch Occup Environ Health.

[9] Khlat M, Ravaud JF, Brouard N, Chau N, Lorhandicap G (2008). Occupational disparities in accidents and roles of lifestyle factors and disabilities: a population-based study in North-Eastern France. Public Health.

[10] The National Research Center for the Working Environment. Tal og fakta om arbejdsmiljøet i Danmark - Arbejdsmiljøprofil i 2018 for brancherne anlægsarbejde, færdiggørelse af byggeri samt opførsel og nedrivning af byggeri [Numbers and facts about the work environment in Denmark - Profile on the Work Environment in 2018 for industries concerning construction work]. [Internet]. Available from: https://arbejdsmiljodata.nfa.dk. Cited February 28, 2020.

[11] Lesuffleur T, Chastang JF, Sandret N, Niedhammer I (2015). Psychosocial factors at work and occupational injury: results from the French national SUMER survey. J Occup Environ Med.

[12] Niedhammer I, Chastang JF, David S (2008). Importance of psychosocial work factors on general health outcomes in the national French SUMER survey. Occup Med (Lond).

[13] Chau N, Bhattacherjee A, Kunar BM, Lorhandicap G (2009). Relationship between job, lifestyle, age and occupational injuries. Occup Med (Lond).

[14] Swaen GM, van Amelsvoort LP, Bultmann U, Slangen JJ, Kant IJ (2004). Psychosocial work characteristics as risk factors for being injured in an occupational accident. J Occup Environ Med.

[15] Sakurai K, Nakata A, Ikeda T, Otsuka Y, Kawahito J (2013). How do employment types and job stressors relate to occupational injury? A cross-sectional investigation of employees in Japan. Public Health.

[16] Åkerstedt T, Knutsson A, Westerholm P, Theorell T, Alfredsson L, Kecklund G (2004). Mental fatigue, work and sleep. J Psychosom Res.

[17] Bláfoss R, Sundstrup E, Jakobsen MD, Brandt M, Bay H, Andersen LL (2019). Physical workload and bodily fatigue after work: cross-sectional study among 5000 workers. Eur J Pub Health.

[18] Swaen GM, Van Amelsvoort LG, Bultmann U, Kant IJ (2003). Fatigue as a risk factor for being injured in an occupational accident: results from the Maastricht cohort study. Occup Environ Med.

[19] Kiconco A, Ruhinda N, Halage AA, Watya S, Bazeyo W, Ssempebwa JC (2019). Determinants of occupational injuries among building construction workers in Kampala City. Uganda BMC Public Health.

[20] Ghosh AK, Bhattacherjee A, Chau N (2004). Relationships of working conditions and individual characteristics to occupational injuries: a case-control study in coal miners. J Occup Health.

[21] Garbarino S, Guglielmi O, Sanna A, Mancardi GL, Magnavita N (2016). Risk of occupational accidents in workers with obstructive sleep apnea: systematic review and Meta-analysis. Sleep.

[22] Yoon JH, Roh J, Kim CN, Won JU (2016). The risk of occupational injury increased according to severity of noise exposure after controlling for occupational environment status in Korea. Noise Health.

[23] Norheim KL, Samani A, Bønløkke JH, Omland Ø, Madeleine P (2020). Physical performances show conflicting associations in aged manual workers. Sci Rep.

[24] Norheim KL, Samani A, Bønløkke JH, Omland Ø, Madeleine P (2019). The effects of age and musculoskeletal pain on force variability among manual workers. Hum Mov Sci.

[25] Baidwan NK, Gerberich SG, Kim H, Ryan A, Church T, Capistrant B (2019). A longitudinal study of work-related psychosocial factors and injuries: implications for the aging United States workforce. Am J Ind Med.

[26] Norheim KL, Samani A, Bønløkke JH, Omland Ø, Madeleine P (2019). Physical-work ability and chronic musculoskeletal complaints are related to leisure-time physical activity: cross-sectional study among manual workers aged 50-70 years. Scand J Public Health.

[27] The National Research Center for the Working Environment (2019). Fakta om Arbejdsmiljø og Helbred 2018 [Facts about Work Environment and Health 2018].

[28] The National Research Center for the Working Environment (2012). Arbejdsmiljø og helbred i Danmark - Spørgeskemaundersøgelse 2012 [Work Environment and Health in Denmark - Questionnaire Survey 2012].

[29] Pejtersen JH, Kristensen TS, Borg V, Bjorner JB (2010). The second version of the Copenhagen psychosocial questionnaire. Scand J Public Health.

[30] De Vet HCW, Terwee CB, Mokkink LB, Knol DL (2011). Measurement in medicine: a practical guide.

[31] Szklo M, Nieto FJ (2019). Epidemiology: beyond the basics.

[32] Bhattacherjee A, Chau N, Sierra CO, Legras B, Benamghar L, Michaely JP (2003). Relationships of job and some individual characteristics to occupational injuries in employed people: a community-based study. J Occup Health.

[33] Kouvonen A, Kivimaki M, Oksanen T, Pentti J, De Vogli R, Virtanen M (2013). Obesity and occupational injury: a prospective cohort study of 69,515 public sector employees. PLoS One.

[34] Gauchard GC, Mur JM, Touron C, Benamghar L, Dehaene D, Perrin P (2006). Determinants of accident proneness: a case-control study in railway workers. Occup Med (Lond).

[35] Van den Berg TI, Elders LA, de Zwart BC, Burdorf A (2009). The effects of work-related and individual factors on the work ability index: a systematic review. Occup Environ Med.

[36] Vandervoort AA (2002). Aging of the human neuromuscular system. Muscle Nerve.

[37] Nelson HD, Nevitt MC, Scott JC, Stone KL, Cummings SR (1994). Smoking, alcohol, and neuromuscular and physical function of older women. Study of osteoporotic fractures research group. JAMA.

[38] Roos E, Bliddal H, Christensen R, Hartvigsen J, Mølgaard C, Søgaard K (2015). Forebyggelse af skader og sygdomme i muskler og led [Injury and disease prevention in muscle and joints].

[39] Chau N, Wild P, Dehaene D, Benamghar L, Mur JM, Touron C (2010). Roles of age, length of service and job in work-related injury: a prospective study of 446 120 person-years in railway workers. Occup Environ Med.

[40] Juliá M, Catalina-Romero C, Calvo-Bonacho E, Benavides FG (2016). Exposure to psychosocial risk factors at work and the incidence of occupational injuries: a cohort study in Spain. J Occup Environ Med.

[41] Rasmussen K, Hansen CD, Nielsen KJ, Andersen JH (2011). Incidence of work injuries amongst Danish adolescents and their association with work environment factors. Am J Ind Med.

[42] Van der Klauw M, Hengel KO, Roozeboom MB, Koppes LL, Venema A (2016). Occupational accidents in the Netherlands: incidence, mental harm, and their relationship with psychosocial factors at work. Int J Inj Control Saf Promot.

