# Supplementary material for: Association of perceived work pace and physical work demands with occupational accidents: a cross-sectional study of ageing male construction workers in Denmark
Source: BMC Public Health. 2022 Jan 6;22:18. doi: 10.1186/s12889-021-12461-6 (PMC8740362; doi:10.1186/s12889-021-12461-6)
Supplement: Supplementary file 1 — Additional file 1: Table S1. Results of the non-response analysis between respondents and non-respondents presented as frequencies and percentages N (%). [file 12889_2021_12461_MOESM1_ESM.docx]

**Association of perceived work pace and physical work demands with occupational accidents: a cross-sectional study of ageing male construction workers in Denmark**

Pernille Weber Hansen^1,2^, Vivi Schlünssen^1,3^, Kirsten Fonager^2,4^, Jakob Hjort Bønløkke^5^,

Claus D. Hansen^6^ and Henrik Bøggild^7^

1) Department of Public Health, Environment, Occupation and Health, Danish Ramazzini Centre, Aarhus University, Denmark

2) Department of Clinical Medicine, Aalborg University, Denmark

3) National Research Centre for the Working Environment, Copenhagen, Denmark

4) Department of Social Medicine, Aalborg University Hospital, Aalborg, Denmark

5) Department of Occupational and Environmental Medicine, Danish Ramazzini Centre, Aalborg University Hospital, Denmark

6) Department of Sociology and Social Work, Aalborg University, Denmark

7) Public Health and Epidemiology Group, Department of Health Science and Technology, Aalborg University, Denmark

***Supplementary Table S1***

***Table S1.*** Results of the non-response analysis between respondents and

non-respondents presented as frequencies and percentages N (%)

|  | Responded on the questionnaire | |
| --- | --- | --- |
|  | No  N (%) | Yes  N (%) |
| Age |  |  |
| 50–54 years | 853 (29.3) | 677 (24.1) |
| 55–59 years | 695 (23.8) | 701 (24.9) |
| 60–64 years | 578 (19.8) | 698 (24.8) |
| 65+ years | 791 (27.1) | 738 (26.2) |
| Residence region |  |  |
| Capital Region of Denmark | 572 (19.7) | 479 (17.0) |
| Region Zealand | 629 (21.6) | 566 (20.1) |
| Region of Southern Denmark | 647 (22.3) | 678 (24.1) |
| Central Denmark Region | 672 (23.1) | 657 (23.4) |
| North Denmark Region | 387 (13.3) | 433 (15.4) |
| Family income^a^ |  |  |
| First quartile | 786 (28.3) | 593 (21.7) |
| Second quartile | 773 (27.9) | 605 (22.1) |
| Third quartile | 640 (23.0) | 738 (27.0) |
| Fourth quartile | 577 (20.8) | 801 (29.2) |
| Number of children |  |  |
| 0 | 2.257 (77.6) | 2.145 (76.3) |
| 1 | 389 (13.4) | 396 (14.1) |
| 2 | 211 (7.3) | 220 (7.8) |
| 3 or more | 50 (1.7) | 52 (1.8) |

^a^ Family income based on the quartiles
